# Current state of the treatment landscape of phenylketonuria

**DOI:** 10.1186/s13023-025-03840-y

**Published:** 2025-06-05

**Authors:** Ine Nulmans, Sien Lequeue, Liesbeth Desmet, Jessie Neuckermans, Joery De Kock

**Affiliations:** Liver Therapy & Evolution Team, In Vitro Toxicology and Dermato-Cosmetology (IVTD) Research Group, Faculty of Medicine and Pharmacy, Vrije Universiteit Brussel, Laarbeeklaan 103, Brussels, B-1090 Belgium

**Keywords:** Phenylketonuria, Phenylalanine hydroxylase deficiency, Newborn screening, Treatment options, Maternal phenylketonuria

## Abstract

**Background:**

Phenylketonuria (PKU) is an inborn error of amino acid metabolism caused by a deficiency of the L-phenylalanine-4-hydroxylase enzyme or its cofactor tetrahydrobiopterin (BH4) resulting in increased levels of phenylalanine (Phe) in blood and cerebrospinal fluid. Symptoms include hypopigmentation, a musty, mouse-like smell and various neurological complications.

**Main text:**

Treatment options include (i) dietary restriction of Phe with supplementation of other amino acids and micronutrients through medical mixtures, (ii) daily dosing of large neutral amino acids, (iii) synthetic forms of BH4 and (iv) bacterial phenylalanine ammonia lyase enzymes. The primary goal of treatment is to lower blood Phe levels and improve quality of life. However, treatment is very demanding for patients as well as their families, and not all treatment options are applicable to every patient.

**Conclusion:**

This review gives a state-of-the-art overview of current treatment options for all PKU patients and additionally speculates on future therapeutic approaches.

## Graphical Abstract

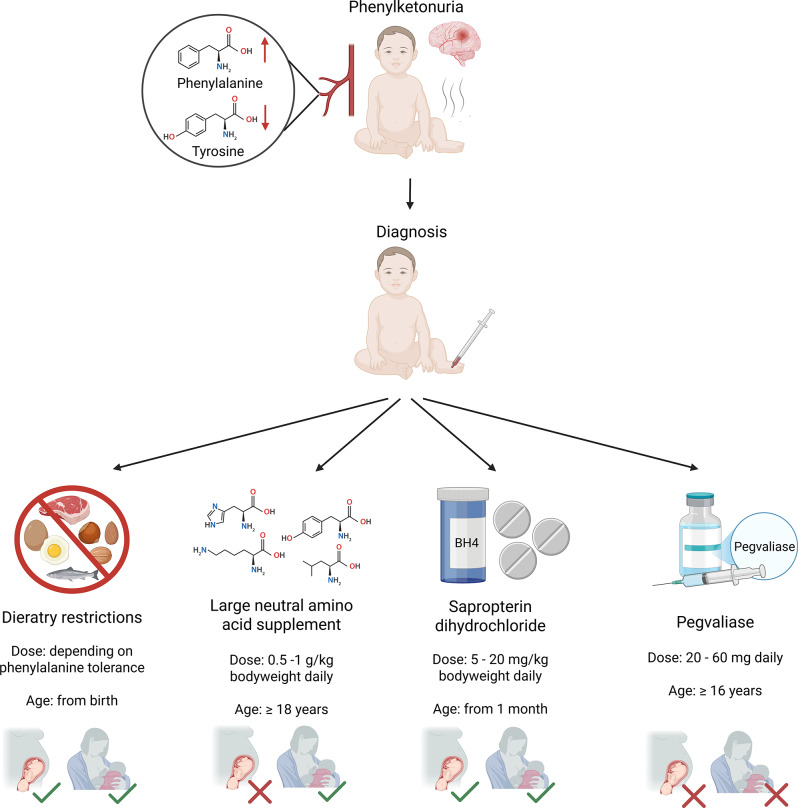

## Background

Phenylketonuria (PKU; OMIM #261600) is an autosomal recessive inborn error of amino acid metabolism, more specifically of phenylalanine (Phe) metabolism [1]. In 1934, PKU was first described by Dr. A. Følling who examined two Norwegian siblings that suffered from severe intellectual disability. Phenylpyruvic acid was detected in their urine which formed phenylketone bodies [2, 3]. The disease is caused by a deficiency in either the L-phenylalanine-4-hydroxylase (PAH) enzyme or, in the minority of cases, its cofactor tetrahydrobiopterin (BH4).

Globally, the prevalence of PKU is 1 in 23,930 newborns with 0.45 million patients affected worldwide [1]. Yet, the prevalence differs significantly among geographical regions. It is the highest in Europe in the Karachay-Cherkess Republic region (Russia; 1 in 850), which can be explained by the high number of consanguineous marriages in this region [4, 5]. Besides, the prevalence rates are notable in Italy (1 in 2700) [5], Ireland (1 in 4500) [5] and Germany (1 in 5360) [1]. However, Western and Southern European countries, including France (1 in 9091), Spain (1 in 10,115) and Belgium (1 in 11,000), showed a lower prevalence [1]. The lowest European PKU rates are observed in the North in Norway (1 in 11,457), Sweden (1 in 12,681) and Finland (1 in 112,000) [1]. PKU prevalences in Canada (1 in 15,000) and the United States of America (1 in 25,000) are slightly higher in comparison with South America (1 in 25,000 to 1 in 50,000) [1, 5, 6]. Some Middle Eastern populations, including Turkey (1 in 4000), Iran (1 in 5000) and Jordan (1 in 5000), showed a prevalence comparable to this of Europe [1, 7]. Lower PKU prevalences are reported in Asian countries including the Philippines (1 in 116,006), Japan (1 in 120,000) and Thailand (1 in 212,535) [1, 6].

This review provides a comprehensive overview of the latest treatment options for the entire PKU patient population including (i) dietary restriction of Phe supplemented with other amino acids and micronutrients through medical formulas, (ii) daily administration of large neutral amino acids, (iii) a synthetic form of BH4 and (iv) bacterial phenylalanine ammonia lyase enzymes. Additionally, it explores potential future therapeutic approaches.

### General symptoms

The PAH enzyme is mainly expressed in hepatocytes and catalyzes the hydroxylation of Phe to tyrosine (Tyr) with the assistance of the BH4 cofactor as well as a non-heme iron and molecular oxygen [8, 9]. A PAH or BH4 deficiency leads to an accumulation of Phe in various body fluids [8, 10]. Additionally, lower Tyr levels are to be expected in patients suffering from PKU as there is an impairment in the conversion of Phe to Tyr [10]. The severity of the disease depends on the residual PAH activity. PKU is classified based on pre-treatment blood Phe concentrations of patients and ranges from mild hyperphenylalaninemia (HPA), with Phe blood concentrations of 120–600 µmol/l, to mild PKU (600–1200 µmol/l) and severe classical PKU (> 1200 µmol/l) [4, 8].

PKU is characterized by hypopigmentation, a musty mouse-like smell and various neurological symptoms including severe intellectual disability, seizures and motor deficits [2, 3, 9]. These symptoms can be explained by the decreased conversion of Phe into Tyr and the accompanying increased Phe and decreased Tyr levels. As melanin pigment is a derivate of Tyr, reduced Tyr levels imply a reduced production of melanin resulting in the hypopigmentation observed in PKU patients [11]. The musty mouse-like smell on the other hand, can be explained by the increased levels of Phe due to which phenylketone bodies, consisting of phenylpyruvate, phenylacetate, phenyllactate and phenylacetylglutamine, are formed [12, 13]. These phenylketone bodies are excreted in patient’s urine and sweat, causing the typical smell [5, 14]. For a long time, it was thought that the neurological symptoms could be explained by the increased levels of Phe and the decreased levels of Tyr and its derivates [15–17]. However, next to the lower blood Tyr levels due to impaired conversion of Phe into Tyr, Phe has a high affinity for the large neutral amino acid (LNAA) transporter, compromising transport of all LNAAs over the blood-brain barrier (BBB), resulting in a reduction of neurotransmitter production [15, 17, 18]. Still, this cannot fully explain the various neurological manifestations and efforts have to be made to better understand the mechanisms behind the neurotoxic effects of increased Phe levels in PKU [19].

Whilst very common in the Pah^enu2^ mouse model for PKU, osteopenia is observed to a lesser extent in human PKU patients [20, 21]. Although studies seem inconclusive concerning the correlation between Phe levels and osteopenia, it is suspected that oxidative stress and alterations in energy homeostasis fulfill an important role herein [20]. Osteoblast differentiation requires an upregulation in oxidative energy production. However, in PKU, a regulator of chromosome condensation 1 deficit with consequent proton leakage is observed, resulting in a reduction of oxidative energy production and increased ROS [20, 22, 23]. As observed in Pah^enu2^ mice, this results in reduced capacity for osteoblast differentiation [20, 21]. Additionally, there was a reduction in gene expression of genes critical to osteoblast function. This hypothesis is confirmed by the administration of antioxidants, resulting in an increase in mitochondrial mass and oxidative energy production leading to improved osteoblast differentiation [20]. Moreover, the reduction in dopamine levels could play a role in PKU induced osteopenia as osteogenic differentiation can be induced by binding of dopamine to dopamine receptors on osteoblasts [21, 24].

Furthermore, it is often difficult to establish a good genotype phenotype correlation in PKU patients as well as to predict their BH4 responsiveness [25]. This could be explained by the large heterogeneity of the disease and the individual metabolism of each patient influences their Phe levels [25, 26]. Besides, 75% of PKU patients are compound heterozygotes, resulting in interallelic complementation of the mutated tetrameric PAH enzyme [19, 27]. Attempts have been made to correlate genotypes to PKU phenotypes based on PAH protein stability, residual enzyme activity and the allelic phenotype value algorithm. Although methods to predict the PKU phenotype improved over the past years, they still do not always correlate with the metabolic phenotype of patients [25, 28]. It is therefore suggested that the pathophysiology of PKU is more complex than initially expected and that more recently discovered neurological processes, including alterations in protein synthesis and energy homeostasis, increased oxidative stress and hypomyelination, could thus complicate the establishment of a good genotype phenotype correlation [15, 16].

### Diagnosis and treatment

In 1953, Bickel et al. successfully treated a 4 year old girl who suffered from PKU [29, 30]. The child was put on a Phe- and Tyr-free diet [29] and within five days, Tyr levels dropped and the child rapidly lost weight while Phe levels were still high. Tyr was thus added to the diet upon which Tyr-levels rose back to normal and the weight loss was temporarily ceased [30]. After three weeks of treatment, general aminoaciduria developed followed by an increase of plasma Phe levels [29, 30]. Additionally, the girl started to steadily lose weight again, seemed unwell and started vomiting frequently, indicating an increase in breakdown of body proteins resulting from a Phe deficiency [30]. Therefore, Phe was added to the diet in a restricted dose matching the daily Phe requirements [29, 30]. This way, one of the cornerstones of the current treatment for PKU, a Phe restricted diet, was developed. Ten years later, in 1963, Guthrie and Susi started screening for PKU by assessing the Phe concentration in a dried blood spot of neonates. This allowed for early diagnosis of the disease and the initiation of the appropriate diet [31].

As treatment plans differ significantly worldwide, there is a need for standardized guidelines. Both American (American College of Medical Genetics and Genomics (ACMG) practice guidelines) and European guidelines have been developed [19, 32]. However, as these diagnosis and treatment guidelines have been established in respectively 2014 and 2017, they do not include treatment with bacterial phenylalanine ammonia lyase (PAL) enzymes and are therefore not completely up to date. Current state of the art diagnosis and treatment for PKU, based on the American and European guidelines but taking into account more recently developed and approved treatment options, are described here.

### Diagnosis

PKU is diagnosed through newborn screening. This implies that newborns are screened early in life through a heel prick after which the dried blood spot is assessed using tandem mass spectrometry (MS/MS) [33, 34]. A robust environment to take blood samples as well as a well-equipped laboratory and proper analysis method are necessary to obtain conclusive results [32]. It is possible to determine increased Phe levels from 24 h after birth. However, it should be taken into account that Phe levels at that point might not have reached their maximum level yet. A more definitive result can be obtained by performing the heel prick 72 h after birth [35]. Cut-off levels for a positive newborn screening test differ widely and range from 65 µmol/l to 234 µmol/l with an average of 130 µmol/l [36]. Upon a positive newborn screening test, PKU should be considered in the differential diagnosis along with a high natural protein intake, general aminoacidemia, liver disease, prematurity and defects in the production or regeneration of BH4 [37, 38]. Patients should then be referred to a specialized metabolic center to properly diagnose PKU by analysis of the complete amino acid panel as well as the Phe/Tyr ratio [39–41]. BH4 deficiency can be excluded by analysis of pterin patterns in patients urine and dried blood spots. Besides, a BH4 loading test can be performed to allow earlier diagnosis and treatment initiation for this group of patients [37, 42]. Once PKU is diagnosed, genotyping might be indicative of a patient’s individual treatment plan [43, 44].

### Treatment initiation

Treatment should be initiated as soon as possible and at least before the age of 10 days [9, 32]. To prevent neurological damage, Phe levels should be within range after the 2nd week of life [45]. There is an absolute consensus that treatment should be initiated in patients with Phe levels > 600 µmol/l. Inconsistent evidence exists about treating patients with blood Phe levels between 360 µmol/l and 600 µmol/l [46, 47]. It is advisable to treat this group of patients until the age of 12 years old after which treatment can be discontinued. Though, it is important to provide sufficient education concerning the risks of maternal PKU and its effects on the fetus in fertile women with Phe levels > 360 µmol/l after the age of 12 years [47–49]. When Phe levels are between 120 µmol/l and 360 µmol/l, no treatment is recommended, nevertheless, patients should be followed up until the age of 2 years to ensure that over time increase of protein intake does not result in increasing Phe levels [47, 48]. The lower blood Phe target is 120 µmol/l as normal growth, protein synthesis and tissue repair cannot be guaranteed below this level. It is advised to ensure that Phe levels do not drop under 30 µmol/l as this increases the risks associated with prolonged low blood Phe levels [19, 50]. The upper Phe level differs with age. Below 12 years, 360 µmol/l is considered the maximum blood Phe level. From 12 years on, Phe levels up till 600 µmol/l are regarded as within range. Higher Phe levels would result in differences in cerebral protein synthesis, changes in white matter and increased oxidative stress [51–53].

The primary goal of treatment is to decrease Phe levels and to obtain normal neurocognitive and psychosocial functioning [19, 32]. Secondary, treatment can increase Phe tolerance and thus improve quality of life and ameliorate symptoms [19]. Treatment for life is recommended and there is no proof that relaxation or ceasing treatment is safe during childhood, adolescence or adulthood [54–57]. Additionally, reinstalling treatment is very challenging due to its impact on patient’s lives [58, 59]. Therefore, lifelong follow-up is recommended to screen for long-term complications, adherence and possibly adjustment of treatment to keep Phe levels within range along with providing appropriate support to both patients and their families [60, 61]. Blood Phe levels are the main follow-up parameter [45, 47, 62]. As blood Phe levels vary during the day with the highest concentration in the morning after overnight fasting, they should be measured at a consistent time, preferably two to three hours after eating and each time, the same method should be used for analysis to allow for an easy comparison of the different measurements. Besides blood Phe levels, the Phe/Tyr ratio might be useful as control parameter. However, it should be measured in the morning after fasting to avoid fluctuations in Tyr levels as these may increase during the day due to Tyr intake. Weekly controls during the first year of life are essential to instruct patient’s parents and obtain full metabolic control [47, 61, 62]. Until the age of 12 years, it is advised to follow-up patients every two weeks. During adolescence, monthly controls are recommended as most patients deviate from treatment during this period [61, 63]. In addition, a shift should be made from informing the parents to informing the patient as this allows for an easier transition from child to adult care. Besides, it leads to more involvement, commitment and responsibility regarding treatment adherence during adolescence and adulthood. It might be advisable to perform neurocognitive evaluations before dietary relaxation due to the increasing upper limit of Phe levels at this age. This to establish a baseline for neurocognitive functioning and to allow proper monitoring during life [61, 64, 65]. As a rule of thumb, it can be stated that the frequency of follow up should be based on the necessary support for each individual patient and their environment as well as on various life changes including change of school, start of employment, moving out and therapy adherence [32, 61].

### Phe restricted diet

The corner stone of PKU treatment consists of a Phe restricted diet. This implies the consumption of low protein foods, containing ≤ 50 mg Phe per g protein, and the administration of a medical mixture to ensure sufficient intake of other amino acids, minerals, fatty acids and vitamins [66, 67]. In newborn children, breastfeeding normally doesn’t cause any problems as it only contains 46 mg Phe per 100 ml milk [32]. Additionally, it ensures normal protein intake without the need for addition of other amino acids, minerals, fatty acids and vitamins. Still, optimal results are obtained when breast milk is alternated with a Phe free infant formula as this results in long-term satisfactory control of Phe levels and growth [68–70]. In general, no more than 20% of energy intake should be consumed under the form of proteins and natural protein intake in adults consists of 6 g per day [71, 72]. However, protein requirements should be individualized and determined on the Phe tolerance of patients which depends on the severity of PKU, the ratio of protein catabolism and protein synthesis, energy intake, patient’s body weight and the target blood Phe concentration [71–73]. Although current evidence is inconsistent, a correlation between obesity or overweight and PKU is assumed, especially in women [74–76]. It is therefore important to determine a patient’s protein need based on ideal body weight and not actual body weight in this group of patients. Calculation based on actual body weight might lead to overestimation of the correct total protein and thus result in excessive Phe levels [32]. In addition rapid growth, changes in body composition and the initiation of additional treatments can influence a patient’s Phe tolerance [77, 78]. Protein intake and Phe tolerance decrease with age, which can be explained by slower growth, and blood Phe levels increase in sick patients as there is a higher energy demand and the catabolism of muscle proteins is elevated during illness [79, 80]. Therefore, these factors should all be considered when determining or adjusting patient’s natural protein intake and treatment. This to ensure that Phe levels stay within range while nutritional requirements are met [77, 80].

Low protein foods can either be natural such as glycomacropeptide or can be modified to contain less protein [67, 81]. Glycomacropeptide contains significant less of the L-amino acids that need to be supplemented including histidine, tryptophan, Tyr and leucine. Additionally, it contains some Phe. Therefore, it is not first choice to complement a Phe restricted diet and a combination of modified low protein foods and a medical mixture to supplement the other amino acids, is more preferable [81]. Modified low protein foods of pasta, bread, cereal and egg and milk replacements already exist [67]. They are produced from food starches such as wheat, potato and maize starch instead of wheat flour. It is important that they do not contain more energy, fat, carbohydrate or sugar than their natural protein containing equivalents [67, 82, 83]. They improve variety of the Phe restricted diet and satisfy the patient’s appetite [67]. Fruit and vegetables contain low levels of Phe and do thus not result in increased Phe levels when consumed in small portions. Therefore, fruits and vegetables containing ≤ 75 mg Phe per 100 g can be consumed without limitations as this allows for a greater dietary variety and improve dietary adherence [81, 84]. As aspartame contains Phe, it should be avoided as much as possible in a Phe restricted diet. However, in case of illness, aspartame containing drugs can be administered if there is no alternative available [85, 86].

### Phe free medical mixtures and LNAAs

As a Phe restricted diet is low in proteins and thus amino acids, Phe free medical mixtures containing L-amino acids should be administered. The addition of such medical mixtures is necessary to allow normal growth and health. They supplement protein, calories and other nutrients [87]. Supplementing Tyr through these L-amino acid supplements is indispensable as less or no Tyr is formed through the hydroxylation of Phe in patients suffering from PKU [88]. Additionally, medical mixtures containing LNAAs including Tyr, histidine, leucine and lysine improve Phe control and tolerance. They improve anabolism and decrease uptake of Phe through the gut due to competition for the LNAA transporter. Through the same mechanism, transport of Phe over the BBB is reduced [89–91]. Administration of LNAAs through medical mixtures increases production of serotonin, norepinephrine and epinephrine. Besides, the competition with Phe for transport over the BBB restores the balance in cerebral protein synthesis [90]. As total protein intake is made up of both natural protein intake and protein intake through medical mixtures, the dosage of the Phe free L-amino acid supplements depends on the natural protein intake. Compared to natural proteins, the efficiency of the supplemented L-amino acids is decreased for which should be compensated when determining the safe level of protein intake for each patient [92]. Some clinical centers administer, besides the LNAAs in the Phe free medical mixtures, an additional dose of 0,5 to 1 g LNAAs per kg bodyweight to treat the excessive Phe levels in adult PKU patients. This resulted in a 40% decrease of blood Phe levels in a single clinical trial [89, 91]. As, to date, there is insufficient evidence concerning the safety and efficacy of the administration of an additional dose of LNAAs and studies only include a small group of patients, it is not formally recommended yet to use LNAAs to treat PKU or to increase dietary Phe tolerance in PKU patients [91, 93, 94]. Besides their functional effects, adverse effects of the L-amino acids should be taken into account as well to establish proper positioning of the medical mixtures and LNAAs. Possible side effects include intestinal problems, caused by the hyperosmolarity of medical mixtures. So far, abdominal pain, constipation and diarrhea have been reported in children. In adults, proteinuria and a decreased glomerular filtration rate can occur due to the lifelong intake of L-amino acid supplements [95–97]. Plasma amino acid levels increase and decrease faster after consumption of medical mixtures containing L-amino acids. Furthermore, oxidation rate is higher compared to natural proteins. It is therefore recommended that medical mixtures are consumed in more frequent but smaller doses. The total intake of medical mixtures should be spread over at least three equal doses during the day. This minimizes fluctuations in Phe levels during the day and results in more optimal blood Phe levels, better dietary tolerance and reduced intestinal problems [92, 98–100].

In addition to lower levels of L-amino acids, Phe restricted diets are low in minerals such as zinc, iron and selenium, vitamins including vitamin B12, fat, trace elements and other nutrients. Patients on such diets are thus at risk for micronutrient deficiencies and low levels of α-linolenic acid, arachidonic acid, eicosapentaenoic acid and docosahexaenoic acid have been observed. Medical mixtures are therefore frequently supplemented with various vitamins, minerals, long chain polyunsaturated fatty acids and carbohydrates to meet nutritional requirements [101, 102]. Low protein diets can also result in lower calcium intake which might add to the osteopenia observed in PKU patients [102]. Sufficient calcium and vitamin D intake as well as frequent physical activity can reduce this risk of osteopenia [103]. Proper assessment of the full nutritional status including a complete plasma amino acid panel and analysis of various vitamins, trace minerals, folic acid, ferritin, hemoglobin, mean corpuscular volume and essential fatty acids should be performed at each follow up. Body mass index and anthropometry can be investigated to complete the nutritional assessment [102]. Patients and their families should be provided with sufficient support regarding the Phe restricted diet and the administration of the bitter-tasting medical mixtures to ensure treatment adherence. Children suffering from PKU are more likely to develop eating disturbances and food neophobia has been observed more frequently in this population. Psychologic support in addition to installing consistent mealtime routines and gradually increasing familiarity with new foods might aid in overcoming these difficulties. Therefore, it is recommended that the team of caregivers consists, besides a metabolic physician specialized in inborn errors of metabolism, of a dietician and a (neuro)psychologist [104–107].

### Sapropterin dihydrochloride

Sapropterin dihydrochloride, the synthetic form of BH4, can be used as treatment in 25–50% of PKU patients, responding to BH4. Decreasing Phe levels are obtained by improved folding and increased stability of the mutated PAH enzyme upon administration of sapropterin dihydrochloride [108]. Often BH4 responsive patients suffer from a mild PAH deficiency or have a mutation in the genes responsible for the production or regeneration of BH4. However, a small number of patients suffering from classical PKU might benefit from sapropterin dihydrochloride treatment as well. BH4 responsiveness is determined through a BH4 loading test and is defined as an increase in natural protein intake ≥ 100% while blood Phe levels remain within range or as a decrease of 30% in Phe levels after a BH4 loading test [109, 110]. Such a test is indicated in every patient unless two null-variants of the PAH enzyme have been identified. Upon a positive BH4 loading test, a treatment trial with sapropterin dihydrochloride is initiated during which its dose is optimized. If no clinical improvement is observed, treatment is discontinued. It should be noted that no BH4 loading test is necessary in patients with a genotype known not to respond to BH4. In patients with a genotype known to be responsive to BH4, a treatment trial might directly be initiated [26, 111, 112]. Both BH4 responsive and unresponsive genotypes are listed in the BIOPKU database [113].

Sapropterin dihydrochloride has recently been approved in children under 4 years of age and no severe adverse effects have been observed [114]. Doses range from 5 to 20 mg per kg bodyweight where 20 mg per kg bodyweight once a day is used as the most common dose. Treatment with sapropterin dihydrochloride increases the Phe tolerance and might thus be useful in patients at the lower end of the PKU spectrum. Dietary Phe tolerance can double or even quadruple while blood Phe levels remain stable [115, 116]. As patient’s Phe tolerance increases, natural protein intake can increase followed by a decrease of the medical mixture intake, taking into account the risk of micronutrient deficiencies, thus requiring sufficient follow up [108]. Altogether, treatment with sapropterin dihydrochloride can significantly improve quality of life in PKU patients and treatment is thus highly recommended in BH4 responsive patients [108, 115, 116].

### Pegvaliase

**Fig. 1:**
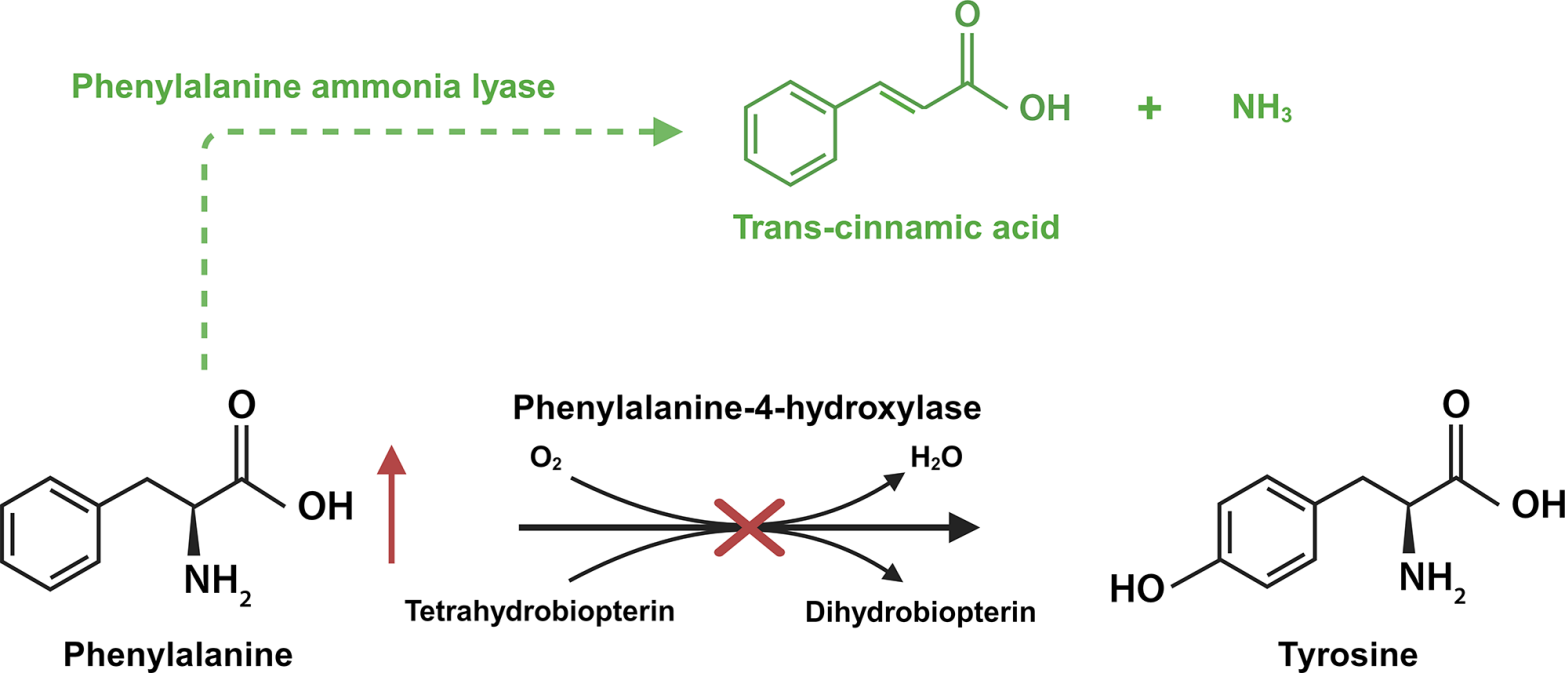
Phenylalanine (Phe) is metabolized to tyrosine by the phenylalanine-4-hydroxylase (PAH) enzyme using molecular oxygen and tetrahydrobiopterin as cofactors. In phenylketonuria (PKU), a PAH deficiency leads to increased levels of Phe causing several symptoms including hypopigmentation and neurological complications. In PKU patients, excessive Phe levels can be decreased by administration of a bacterial phenylalanine ammonia lyase which converts Phe into trans-cinnamic acid through a pathway independent of PAH

As of 2018, the bacterial PAL enzyme from *Anabaena variabilis* (AvPAL) has been approved to treat PKU patients aged 16 years or older with uncontrolled blood Phe concentrations > 600 µmol/l despite a Phe restricted diet [117–119]. The recombinant pegylated AvPAL enzyme, also known as pegvaliase, decreases blood Phe levels through a PAH-independent pathway [120]. As displayed in Fig. 1, it degrades Phe into trans-cinnamic acid and ammonia [121]. The goal of treatment is to relax dietary restrictions as much as possible while blood Phe levels maintain within range. Treatment is considered successful if Phe levels are significantly reduced or if Phe levels stay stable while the diet is normalized. Besides, if patient’s quality of life improves, treatment with the recombinant PAL enzyme can be regarded as clinically meaningful. However, treatment should be ceased at any point but at least within 52 weeks if disease parameters do not ameliorate [122].

Pegvaliase is administered subcutaneously in the thighs or lower part of the abdomen with an initial dose of 2,5 mg every week during four weeks [118, 119]. The initial dose is administered under the supervision of a trained clinician and the injection site should be rotated with each dose [118, 122]. Afterwards, the dose is gradually increased until the maintenance doses between 20 mg to 60 mg injected daily is achieved [118]. The individual maintenance dose can be lower than 20 mg per day and depends for each patient on the tolerability and protein intake [119, 122]. During titration, blood Phe levels of patients might drop < 30 µmol/l in which case natural protein intake should be increased while it might be necessary to decrease the dose of the recombinant PAL enzyme [118]. Frequent monitoring every one to four weeks while initiating treatment allows proper balancing of increased doses of pegvaliase with decreased dietary restrictions. Natural protein intake should be increased with 10 g to 20 g when Phe levels drop < 120 µmol/l. Protein derived from medical mixtures can be reduced equally with regard to the risk of micronutrient deficiencies [122].

Various side effects have been observed with the use of pegvaliase. The most common ones include reactions on the injection site, pruritus, general skin rash, erythema, myalgia, arthralgia, abdominal pain, nausea, vomiting, diarrhea and hypersensitivity. Due to the bacterial origin of recombinant AvPAL, anaphylactic reactions occurred in some patients [118]. Besides adequate information and training concerning the injections of pegvaliase, patients should be informed about the risk of severe hypersensitivity or anaphylactic reactions and should receive sufficient training in recognizing sings of anaphylactic reactions as well as in handling and administering an epinephrine autoinjector [122]. It is advised to administer antihistaminic and antipyretic drugs to reduce the risk of these severe acute adverse events [118, 122]. Furthermore, it is important that there is an observer, trained to recognize and treat acute systemic hypersensitivity or anaphylaxis, who accompanies the patient for minimally one hour post injection and this during at least the first six months from initiation of treatment [118, 122].

### Treatment adherence

Treatment should be maintained throughout life. Upon relaxation of dietary restrictions at the age of 12 years due to the increase of the upper limit for blood Phe levels to 600 µmol/l, treatment adherence might decrease. At this age, there is also a shift from informing the parents and family to informing the patient [61, 65]. Additionally, when patients should transfer from pediatric centers to adult centers, there is often a lost to follow up. This implies that the patients themselves are frequently unaware of the severity of their condition requiring lifelong treatment [123]. In combination with the strict Phe free diet reducing quality of life, this often results in relaxing or even ceasing treatment leading to adverse effects including anxiety, depression and deficits in executive functioning. This has a significant impact on the educational attainment and socioeconomic status of these untreated patients [124–126]. Impaired neurological functioning limits the understanding of the disease even more in untreated PKU patients. As a result, reinstallation of treatment is thus very difficult and regaining metabolic control is complex [127, 128]. This part of the patient population forms a major challenge and proper treatment support during childhood as well as a controlled transfer from pediatric to adult care facilities are necessary to avoid lost to follow up and improve treatment adherence [65, 123]. Adverse effects occurring after treatment relaxation will ameliorate once proper metabolic control is obtained. Moreover, patients who didn’t get access to early treatment might still gain some behavioral improvement with better seizure control and decreased psychiatric symptoms [123, 127]. It is thus worth to install a treatment trial in this group of patients even though symptoms will not completely resolve and no improvement in cognitive abilities is observed. Treatment should be discontinued if there are no beneficial effects [125].

### Maternal PKU

In pregnant women, increased blood Phe levels result in the maternal PKU syndrome. This is characterized by the teratogenic effects of excessive Phe and can lead to physical and cognitive defects in the fetus including microcephaly, intellectual disability, poor growth, congenital heart defects and nonfamilial facial features. Ideally, metabolic control of blood Phe levels is obtained prior to congestion to avoid teratogenic effects on the fetus as the severity of these possible adverse effects correlates to the maternal blood Phe level [129, 130]. It has been shown that the IQ score of offspring decreases with 4,7 points when maternal blood Phe levels increase with 60 µmol/l [49, 131]. As Phe is transported over the placenta, fetal Phe levels are higher compared to maternal blood Phe levels. Therefore, it is recommended that blood Phe levels in pregnant women are < 240 µmol/l to completely circumvent all teratogenic effects. However, especially in the second and third trimester of pregnancy, too low maternal blood Phe levels lead to an increased risk of intrauterine growth restriction [130, 132, 133]. This is associated with increased risks of diabetes, hypertension and other cardiovascular diseases in offspring. Maternal blood Phe levels are therefore regarded as within range between 120 µmol/l and 360 µmol/l [130, 132].

In fertile women with blood Phe levels between 360 µmol/l and 600 µmol/l, it is thus important to provide sufficient information concerning the need for treatment before as well as during pregnancy [134]. Unplanned pregnancies should be anticipated by proper sexual education and explaining the risks of unprotected sexual contacts from the first menstrual cycle on. Weekly monitoring of blood Phe levels is required pre-conception. During pregnancy, even more frequent follow up is necessary to keep Phe levels within range. To avoid effects of either too low or too high blood Phe levels on the fetus, it is advised to see pregnant patients twice a week [135, 136]. Treatment should be initiated pre-conception or at least as early as possible during pregnancy. A Phe restricted diet is initiated and BH4 responsive patients can continue their treatment with sapropterin dihydrochloride during pregnancy [136, 137]. Treatment with sapropterin dihydrochloride can furthermore be initiated during pregnancy in untreated patients with blood Phe levels between 360 µmol/l and 600 µmol/l, yet, it is important that BH4 responsiveness is tested prior to conception to allow correct interpretation of these test results [137]. Administration of LNAAs is not recommended in pregnant women due to the lack of information of their effect on fetal growth and neurological development. Besides, they do not lower Phe levels sufficiently to exclude teratogenic effects on the fetus and safety data during pregnancy is lacking [81, 138]. Due to insufficient data regarding the safety of treatment with pegvaliase during pregnancy, it is advised to cease treatment with the bacterial PAL enzyme 4 weeks prior to conception. A Phe restricted diet should be initiated while the start-up of sapropterin dihydrochloride therapy can be considered in these patients [122].

During pregnancy, extra vigilance is indicated when installing a Phe restricted diet. Pregnant women are at higher risk for micronutrient deficiencies and vitamin and mineral intake should be cautiously monitored [139, 140]. They should be informed about the risk of Phe over restriction. Moreover, there is a risk for too low intake of food derived folic acid and vitamin B12. Folic acid should be administered in a dose of 0,4 mg per day on top of the folic acid supplemented in the medical mixtures to decrease the risk of neural tube defects [140–142]. As a vitamin B12 deficiency can, next to elevated Phe levels, induce congenital heart disease in the fetus, oral or intramuscular supplementation might be necessary to decrease the risk of this adverse effect [140, 142, 143]. Additionally, caution should be paid to excessive vitamin A intake, derived from the medical mixtures, as this poses a risk of teratogenic effects on the fetus [87]. As stated before, Phe restricted diets are low in arachidonic acid and docosahexaenoic acid. Both are however essential for fetal growth and pregnant women are advised to increase the intake of docosahexaenoic acid with 200 mg per day. If supplementation through the medical mixtures proofs insufficient or in case of a biochemical deficiency, it might be necessary to administer an additional dose [144–147].

Energy needs increase in pregnant women. Contradictory, energy intake tends to lower in PKU women, mainly in the first trimester of pregnancy, leading to weight loss and increased blood Phe levels. Numerous explanations have been proposed including morning sickness with nausea and vomiting, poor adherence to the medical mixtures, dislike of low protein foods and the inability to prepare low protein meals [133, 148]. As maternal weight loss and lower energy intake have been associated with increased risk of microcephaly, decreased fetal growth and lower birth weight, sufficient follow up is necessary and in some cases, monitoring off the whole nutritional status is advised, especially when adherence to treatment is suboptimal [148–150]. To provide for the increased protein need during pregnancy, it is recommended that the total protein intake is increased to ≥ 70 g per day. Moreover, Phe requirements increase due to the fetal-maternal anabolism, particularly in the second and third trimester of pregnancy when the fetus does not suffer from PKU. To decrease the risk of prolonged too low maternal Phe levels and its effects on the fetus, maternal Phe intake should be increased with 50 to 100 mg per day. Increasing protein and Phe requirements during each trimester should be taken into consideration when determining the total protein intake for each individual patient [19, 135, 150, 151].

Besides monitoring of the nutritional status and blood Phe levels of the mother, it is important to carefully monitor the fetus during pregnancy. Monitoring should start at the beginning of pregnancy and should continue at least until the end of organ development at 18 to 22 weeks [152–154]. Fetal growth must be followed up more strictly, especially when maternal blood Phe levels are not within range. More frequent ultrasound examinations might be necessary to avoid later onset of growth retardation and microcephaly as well as to screen for cardiac deficits and fetal anomalies [154, 155]. Postpartum, offspring of PKU mothers should be followed up for possible birth defects and their general development. PKU mothers should then return to their standard treatment from before conception [135, 156]. This with the exception of breastfeeding patients treated with pegvaliase as there is no data available of the presence of the bacterial PAL enzyme in the breast milk nor of its effect on the newborn [122]. Although treatment with sapropterin dihydrochloride is no contraindication for breastfeeding, it is advised to be cautious as only limited data is available. So far, no effects have been observed in breast fed newborns from PKU mothers treated with sapropterin dihydrochloride [157]. PKU mothers are encouraged to breast feed. Possible excessive Phe levels in their breast milk do not cause any problem for their non PKU offspring as they possess an unaffected PAH enzyme [158, 159]. However, as breastfeeding requires a higher calorie intake from the PKU mother, it is necessary to adjust the total protein intake accordingly. An increase of 15 g protein per day is recommended. Post lactation, PKU mothers should return to their regular dietary restrictions [19, 159, 160].

A general overview of the various treatment options for the entire patient population can be found in Table 1.

**Table 1:** Overview of the different treatment options for PKU

	Phe restricted diet and medical mixtures	LNAAs	Sapropterin dihydrochloride	Pegvaliase
Mechanism of action	Decreasing Phe intake while administering the other L-amino acids and essential micronutrients through medical mixtures.	Reducing the uptake of Phe in the gut by competition for the LNAA transporter. Transport over the BBB is reduced in the same manner.	Improved folding and stabilizing of the mutated PAH enzyme.	Decreasing Phe levels through a PAH-independent pathway where Phe is converted into trans-cinnamic acid and ammonia.
Treatment goal	Lowering blood Phe levels.	Lowering blood Phe levels and increasing dietary Phe tolerance.	Lowering blood Phe levels and increasing dietary Phe tolerance.	Lowering blood Phe levels while normalizing the diet.
Dose	Total protein intake depends on Phe tolerance and various factors such as age, severity of PKU, body weight, body composition, energy intake, general health and pregnancy should be considered.	An additional oral dose of 0,5 to 1 g LNAAs per kg bodyweight.	Oral treatment of 5 to 20 mg per kg bodyweight once a day.	Subcutaneous injection of 20 to 60 mg once a day.
Patient population	From birth	≥ 18 years	≥ 1 month in BH4 responsive patients	≥ 16 years
Pregnancy	Yes	Not recommended	Yes in BH4 responsive patients	No data regarding the safety during pregnancy.
Breastfeeding	Yes	Yes	Yes in BH4 responsive patients	No data regarding the safety during breastfeeding.
Advantages	- Can be used in all patients.	- Increases dietary Phe tolerance thus improving quality of life.	- Can be used in very young patients. - Increases dietary Phe tolerance thus improving quality of life. - No serious adverse effects have been reported.	- Can be used in patients who do not respond to the Phe restricted diet. - Normalization of diet improves quality of life.
Disadvantages	- Adverse effects including abdominal pain and proteinuria caused by the medical mixtures. - Poor adherence due to the strict dietary restrictions and bitter taste of the medical mixtures.	- Insufficient data regarding its safety and efficacy so formal use has not yet been recommended. - Adverse effects including proteinuria and intestinal problems such as abdominal pain and diarrhea.	- Can only be used in 25–50% of the patient population.	- Patients should be supervised 1 h post injection. - Premedication is required. - Serious adverse effects including anaphylactic shock can occur.

### Future perspectives

Although treatment options improved compared to PKU treatment in 1953, the currently available treatment strategies still have their shortcomings as it is very demanding to adhere to a Phe restricted diet [19, 161]. Moreover, treatment with sapropterin dihydrochloride can only be administered in a limited patient population [162] and pegvaliase is prone to elicit an immune response despite its pegylation, resulting in a higher clearance and a very unpredictable efficacy of the drug [119, 163]. Therefore, it is necessary to research and develop alternative strategies to treat PKU. Indeed, as mentioned in the ACMG practice guidelines of 2014 for the treatment of PKU, there is a need for new therapies that not only lower blood Phe levels in patients, but also significantly improve quality of life for both patients and their families [19].

In analogy to treatment with sapropterin dihydrochloride, a new therapy containing sepiapterin, a natural precursor to the BH4 cofactor, is under development [5, 164–166]. Sepiapterin has a higher capacity to enter the cell which results in higher BH4 levels in both blood and cerebrospinal fluid compared to treatment with sapropterin dihydrochloride [164, 165]. This could imply that a bigger patient population might benefit from the administration of a daily dose sepiapterin of 20 to 60 mg per kg bodyweight [5]. Results of the phase 3 clinical trial (NCT05099640) are promising and show a reduction of ≥ 30% in blood Phe levels without any severe adverse effects [166, 167]. It is expected that marketing authorization for treatment with sepiapterin will be sought in the near future as the phase 3 clinical trial has been finished successfully [167]. Hence, it should be noted that there is a significant probability of treatment response in only a small group of PKU patients upon administration of sepiapterin. This due to the similar mechanism of action as sapropterin dihydrochloride, thus implying the need for sufficient residual activity of the mutated PAH enzyme. A sepiapterin-based treatment could thus provide an additional treatment option, yet, it does not aid in broadening treatment options to cover the total PKU patient population.

To overcome the immunogenic reactions against treatment with pegvaliase, the bacterial PAL enzyme can be encapsulated into red blood cells derived from an O-negative donor. This way, the red blood cells shield the PAL enzyme from anti-PAL antibodies [5, 168]. Upon transfusion of the PAL containing red blood cells and entry of Phe into the cell, Phe is metabolized into trans-cinnamic acid and ammonia [5]. Treatment with in red blood cells encapsulated PAL has been studied in Pah^enu2^ mice, the murine model for human PKU, and proved able to reduce blood Phe until levels within range are obtained [168, 169]. Weekly dosing is necessary to maintain blood Phe levels < 360 µmol/l [5, 169]. A clinical trial to test the efficacy of PAL-containing red blood cells (NCT04110496) was initiated, however, the trial was terminated as the company funding the trial shifted its focus into developing novel cancer treatments [167]. Further research is thus necessary to investigate if treatment with PAL-containing red blood cells could be successful in PKU patients. However, even if this cell therapy proves successful in clinical trials, the applicability under its current form will be limited due to the scarcity of red blood cells derived from an O-negative donor. It therefore might be necessary to reform the treatment towards a more personalized therapy where the blood donor and PKU patient are matched as is currently done with blood transfusions as well as organ donation in order to allow broader applicability of this treatment approach.

In addition, completely new treatment options are being investigated. Studies have shown that administration of antioxidants including L-carnitine, melatonin, vitamin E and ascorbic acid can reduce oxidative stress in both a rat model for PKU and PKU patients. Although antioxidant therapy cannot be harnessed as stand-alone treatment, it is hypothesized that the addition of an antioxidant to the already established individualized treatment plan can ameliorate PKU symptoms, even in early treated patients with late-onset neurological complications [170, 171].

Additionally, to date, there is no treatment available that corrects the metabolic block in PKU. A PAH gene therapy could rectify the deficient PAH enzyme and restore the Tyr production from Phe in PKU patients. Besides, it would allow a normalized diet, improving quality of life [5, 172]. Preclinical studies with adeno-associated viral vectors (AAVs) containing the PAH gene, proved able to obtain metabolic control in Pah^enu2^ mice [173, 174]. Three clinical trials (NCT03952156, NCT04480567 and NCT06061614) are testing AAV-PAH therapy in PKU patients. However, study results are not yet available [167]. Besides, deployment of adenoviral vectors and AAVs is not without risks. It incorporates the possibility of an immune response against the viral vectors, either due to previous natural exposure or caused by multiple treatment injections, resulting in reduced treatment efficacy [5, 172]. Additionally, hepatocyte proliferation eventually leads to decreased PAH expression due to loss of the AAV-PAH gene therapy and liver tumors, caused by insertional mutagenesis, have been observed after administration of AAV therapy in mice [172]. As there is no pre-existing immunity against lentiviral vectors, they could provide a solution to this problem. Moreover, administration of lentiviral vectors results in genomic integration of the PAH gene, overcoming the problems caused by hepatocyte turnover. Nevertheless, the risk of insertional mutagenesis remains [5] and production of sufficient quantities of the lentiviral vector for the treatment of human livers, is challenging [175]. The debate whether or not to administer viral vector based gene therapy in humans thus remains [172]. Still, PAH gene therapy could completely restore the metabolic degradation pathway of Phe and simultaneously ensure a quality of life for PKU patients comparable to this of the healthy population. Further investigations into the administration of such a gene therapy are thus indispensable as they might broaden treatment options for the total patient population while diminishing the burden associated with the disease.

mRNA therapy, where the PAH mRNA is encapsulated in lipid nanoparticles (LNP), could provide another route to restore PKU metabolism [172]. By using liver specific LNPs, the PAH mRNA is taken up into the hepatocyte followed by translation into the PAH enzyme [5]. Successful proof of principle studies have already been conducted in the Pah^enu2^ mouse model [176, 177]. One clinical trial (NCT06147856) to assess the safety and tolerability of such a PAH mRNA treatment has been launched but patient enrollment has not yet started [167]. PAH mRNA therapy could overcome some of the hurdles encountered with gene therapy. As there is no genomic integration, there is no risk for insertional mutagenesis or hepatocellular carcinoma. However, the short half-life of mRNA therapy is a drawback resulting in the need for more frequent administrations [5, 172]. More data is needed to determine its therapeutic effect and to establish the position of mRNA-based therapy in the context of PKU.

Orthotopic liver transplants can restore PAH deficiency as well. However, despite its clinical acceptance, there is a shortage of transplantable grafts, making it only a limited treatment option for PKU patients [178]. Therefore, hepatocyte transplantation followed by liver repopulation is being investigated as a new cell-based treatment for PKU [172]. As sufficient liver repopulation with these transplanted hepatocytes might be a drawback, hepatocytes from a healthy donor have been genetically modified using CRISPR-Cas to become cytochrome p450 reductase (Cypor) deficient [172, 179]. As Cypor is required to metabolize acetaminophen into its hepatotoxic metabolite N-acetyl-p-benzoquinone imine, Cypor deficient hepatocytes are insensitive to acetaminophen induced toxicity [179]. Post-hepatocyte transplantation, acetaminophen is then administered, inducing hepatotoxicity and resulting in selection of and liver repopulation with the transplanted Cypor deficient hepatocytes. This results in sufficient liver repopulation with hepatocytes expressing an active PAH enzyme [175]. Complete metabolic control has been obtained after a Cypor deficient hepatocyte transplant in the murine PKU model. This resulted in decreased blood Phe levels while still maintaining functional cytochrome p450 metabolism without serious adverse effects [175, 179]. Immunosuppressive treatment however is still pivotal to avoid rejection of the transplanted hepatocytes [178] and although preclinical studies seem promising, more data and research, with regard for possible ethical concerns about the use of CRISPR-Cas in humans, is necessary to assess the potential of Cypor deficient hepatocyte transplantation in PKU patients.

## Conclusion

Current treatment options for PKU, including a Phe-restricted diet, sapropterin dihydrochloride, LNAA’s and pegvaliase, are often very demanding and not yet completely ideal as they do not cover the complete patient population. Therefore, research into alternative new therapies that not only reduce blood Phe levels, but also improve quality of life, is needed to minimize the burden of the disease. Although efforts are being made to meet this need for new treatment options, especially by researching the possibility to deploy advanced therapy medical products, including cell- and gene-therapies, hereby covering the total patient population, often more data is needed to properly establish their use and implementation in the treatment guidelines for PKU.

## Data Availability

No new data was generated during the course of writing this comprehensive review. All information was obtained from publicly available scientific literature.

## Abbreviations

AAVs: Adeno-associated viral vectors
AvPAL: *Anabaena variabilis* phenylalanine ammonia lyase
BBB: Blood-brain barrier
BH4: Tetrahydrobiopterin
Cypor: Cytochrome p450 reductase
HPA: Hyperphenylalaninemia
LNAA: Large neutral amino acid
LNP: Lipid nanoparticles
MS/MS: Tandem mass spectrometry
PAH: L-phenylalanine-4-hydroxylase
PAL: Phenylalanine ammonia lyase
Phe: Phenylalanine
PKU: Phenylketonuria
Tyr: Tyrosine
